# Analysis of human neutrophil phenotypes as biomarker to monitor exercise‐induced immune changes

**DOI:** 10.1002/JLB.5A0820-436R

**Published:** 2020-09-06

**Authors:** Roy Spijkerman, Lillian Hesselink, Carlo Bertinetto, Coen CWG Bongers, Falco Hietbrink, Nienke Vrisekoop, Luke PH Leenen, Maria TE Hopman, Jeroen J Jansen, Leo Koenderman

**Affiliations:** ^1^ Department of Trauma Surgery University Medical Center Utrecht Utrecht The Netherlands; ^2^ Center for Translational Immunology (CTI) University Medical Center Utrecht Utrecht The Netherlands; ^3^ Department of Respiratory Medicine University Medical Center Utrecht Utrecht The Netherlands; ^4^ Institute for Molecules and Materials (Analytical Chemistry) Radboud University Nijmegen The Netherlands; ^5^ Department of Physiology Radboud Institute for Health Sciences (RIHS) Radboud university medical center Nijmegen The Netherlands

**Keywords:** innate immune system, neutrophil, prolonged walking, repetitive exercise

## Abstract

The amplitude of the innate immune response reflects the degree of physiological stress imposed by exercise load. An optimal balance of exercise intensity and duration is essential for a balanced immune system and reduces the risk of dysfunction of the immune system. Therefore, it is hypothesized that neutrophils, as key players in the innate immune system, can be used as biomarker in detecting overtraining. The aim was to monitor the state of the innate immune system by phenotyping neutrophils during consecutive bouts of prolonged exercise. Study subjects were recruited from a cohort of walkers participating in a walking event on 3 consecutive days. Participants with immune deficiencies were excluded. Questionnaires to determine the physiological status of the participants were completed. Analysis of neutrophil receptor expression was done by a point‐of‐care fully automated flow cytometer. A total of 45 participants were recruited, of whom 39 participants were included for data analysis. Study participants had a median age of 64 (58‐70) years. The absolute numbers CD16^dim^/CD62L^bright^ and CD16^bright^/CD62L^dim^ neutrophils were increased after the first 2 days of exercise followed by an adaptation/normalization after the third day. Participants with activated neutrophils (high CD11b expression) had an impaired physical feeling indicated by the participant on a lower visual analog scale compared to participants who did not have activated neutrophils (*P* = 0.017, *P* = 0.022). Consecutive days of prolonged exercise results in an initial systemic innate immune response, followed by normalization/adaptation. Increased neutrophil activation was associated with impaired physical feeling measured by a validated VAS score indicated by the participant. Fully automated point‐of‐care flow cytometry analysis of neutrophil phenotypes in a field laboratory might be a useful tool to monitor relevant differences in the systemic innate immune response in response to exercise.

## Graphical Abstract

Fully automated flow cytometry analysis of neutrophils obtained in a field laboratory after repetitive prolonged exercise.

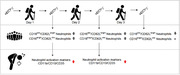

**Abbreviations**
ANOVA: analysis of variance
BMI: body mass index
CD: cluster of differentiation
CR: complement receptor
DBP: diastolic blood pressure
HR: heart rate
IQR: interquartile range
MFI: median fluorescent intensity
SBP: systolic blood pressure
VAS: visual analog scale
WBC: white blood cell count

## 1 INTRODUCTION

The immune system is very responsive to exercise, which leads to specific responses to acute and chronic exercise.^1^, ^2^, ^3^ The amplitude of the immune response reflects the degree of physiological stress imposed by the exercise workload.^2^, ^3^, ^4^ Multiple studies have linked excessive physical activity to transient immune dysfunction and an increased risk of developing infections.^5^, ^6^, ^7^, ^8^, ^9^ In contrast, normal physical activity and regular sports are related to a reduction of infections and other diseases.^10^, ^11^, ^12^ It has been hypothesized that the relationship between absolute exercise load and risk of illness is a J‐shaped curve, with very low or no training being associated with a higher risk of illness, moderate training associated with low illness risk and very high training associated with the highest risk of acute illness.^13^ This suggests that an optimal balance in exercise intensity and duration is essential for a balanced immune system.^14^ Therefore, a biomarker that can be used to monitor the immune system during and after repetitive bouts of prolonged exercise may help to determine the optimal exercise load and thus training program.^15^

Many studies showed an altered adaptive immunity (T‐cell changes, NK cell activity, salivary IgA production), cytokine production and innate immunity (granulocyte cell count, granulocyte respiratory burst, neutrophil/lymphocyte ratio, and Mϕ activity) for several hours to days during recovery from prolonged exercise.^16^, ^17^, ^18^, ^19^, ^20^ The innate immune system demonstrates the most pronounced changes after prolonged exercise.^17^ The primary effector cells of the innate immune system are granulocytes and monocytes.^21^, ^22^, ^23^ Receptor expression on these cells, measured by flow cytometry, has proven to give insight into the status of the innate immune system.^24^, ^25^, ^26^

A recent study showed that intensive exercise led to the mobilization of neutrophil phenotypes associated with systemic inflammation and immunosuppression.^27^ After repetitive prolonged exercise on consecutive days the neutrophil phenotypes, CD16^dim^/CD62L^bright^ and CD16^bright^/CD62L^dim^ increase in the peripheral blood.^27^, ^28^ The CD16^dim^/CD62L^bright^ cells are young neutrophils with a band shape nucleus, most likely recruited from the bone marrow.^29^ The CD16^bright^/CD62L^dim^ cells consist of mainly neutrophils containing hyper‐segmented nuclei that show a more activated and immunosuppressive phenotype.^28^ The presence of this immunosuppressive phenotype during exercise might be involved in the increased risk of developing infections.^27^, ^28^ Neutrophil receptor expression can, therefore, be a possible biomarker in monitoring the balance of the immune system during and after repetitive, prolonged exercise.^24^

Analysis of neutrophil receptor expression used to have many limitations that precluded broad application in a non‐laboratory setting. These limitations include a short time window‐of‐opportunity for analysis, because inflammatory cells are easily activated by ex vivo manipulation and flow cytometry requires a fully functional immunological laboratory with experienced laboratory personnel.^25^, ^30^ The technique of fully automated flow cytometry has become available that circumvents most of these limitations and is applicable in a field laboratory setting.^25^, ^30^ This now allows analysis of the immune system in athletes at the sports site.

To get more insight into the potential role of neutrophil phenotypes in the exercise‐induced immune response, we investigated neutrophils as read out for the innate immune system point‐of‐care during repetitive, prolonged exercise in a large‐scale public walking event where athletic accomplishment of distance goals is closely monitored. The primary aim of our study was to find a biomarker that is associated with overreaching. Our secondary study goal was to study neutrophil kinetics during repetitive, prolonged walking.

## 2 MATRIALS AND METHODS

### 2.1 Study design

Subjects were recruited from a cohort of participants in the Nijmegen 4 Day Marches that filled out a questionnaire as part of the Nijmegen Exercise Study (http://www.vierdaagseonderzoek.nl/en/). To investigate the immune response in the general population undertaking moderate exercise, only participants with immune deficiencies or taken immune suppressive therapies were excluded. A total of 45 participants were included in this study. Three participants dropped out of the study after 1 day of walking, 1 participant dropped out after 2 days and 1 participant did not show up at day 2 of walking. During blood analysis, one participant appeared to be FcγRIII (CD16)‐deficient and was, therefore, excluded as well. As a result, a total of 39 participants were included for data analysis. All participants completed a distance of 30, 40, or 50 km/day on three consecutive days at a self‐selected pace. Every participant was assigned to an individual distance (30, 40, or 50 km) and completed the same distance on the three consecutive exercise days. The study was approved by the Medical Ethical Committee of the Radboud University Medical Center under protocol number CMO‐nr 2007–148 and all participants gave written informed consent prior to participation. All procedures performed in this study were in accordance with the 1964 Helsinki declaration and its later amendments.

### 2.2 Baseline measurements

**FIGURE 1:**
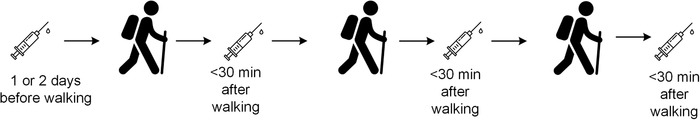
**Design of the study**. Baseline data were collected 1 or 2 days before the start of the event and each day of walking within 30 min after completion of the exercise

Baseline data were collected 1 or 2 days before the start of the event, after a minimum resting period of 24 h (Fig. 1). A period of 24 h recovery was based on the kinetics of immune cell count recovery post‐exercise.^4^ At baseline, body height and weight (Seca 888 scale, Hamburg, Germany) were measured to calculate body mass index (BMI). Waist circumference was measured with a measuring tape (Seca 201, Chino, CA, USA). Resting heart rate (HR), systolic blood pressure (SBR), and diastolic blood pressure (DBP) were measured in the supine position after a 5 min resting period. All participants completed a general questionnaire on demographics, level of education, smoking, and medication use. Heart rate (HR) was used to estimate exercise intensity as a percentage of the maximum HR (exercise intensity = measured HR/expected maximal HR × 100%, where expected max HR = 208 –132 (0.7 × age)).^31^ Exercise intensity was determined using the guideline of the America Heart Association.^32^ Performing 50–70% of your maximum heart rate is considered moderate intensity. From 70% to 85% of your maximum heart rate is regarded as vigorous intensity. For determination of the exercise intensity, heart rate at day 1 was measured during the prolonged exercise at every 5‐km checkpoint. The mean heart rate per person was used for exercise intensity calculation.

### 2.3 Visual Analog Scale questionnaire

At baseline, and after every day of walking exercise, participants were instructed to score their physical feeling, exercise effort, and perceived muscle pain using the Visual Analog Scale (VAS), which consists of a horizontal line of 100 mm.^33^ This method has been extensively validated and used in many studies.^34^, ^35^, ^36^ The aspect of physical feeling was determined with the question: “Show with a vertical line how you feel today,” bad feeling (0 mm) to best feeling (100 mm). The exercise effort was determined with the question: “Show with a vertical line how much effort the exercise costed you today,” it felt like a short walk (0 mm) to it felt like an extremely long walk (100 mm). The aspect perceived muscle pain was determined with the question, “Show with a vertical line how many muscle complaints you had today,” no complaints (0 mm) to worst pain ever (100 mm). Furthermore, participants were asked whether they took painkillers after every day of walking.

### 2.4 Blood sampling

Venous blood was drawn at baseline and after each day of walking <30 min after completion of the exercise (Fig. 1). A 4 ml Vacutainer^®^ sodium heparin blood tube (Becton Dickinson, Oakville, ON, USA) was drawn that was directly inserted in the automated AQUIOS CL^®^ “Load & Go” flow cytometer (Beckman Coulter, Miami, FL, USA).

### 2.5 Flow cytometry analysis

The AQUIOS CL^®^ combines robotic automated sample preparation with analysis of cells using flow cytometry.^25^ The AQUIOS CL^®^ has one 488 nm diode laser, 2 light scatter channels (forward scatter and side scatter), 5 fluorescence channels, and an electronic volume measure. Absolute leukocyte count was based on an electronic‐volume measurement. A cassette filled with blood tubes was placed in the machine and the barcodes of the samples were saved. After automatic blood mixing, the samples were cap‐pierced, and 43 μl was pipetted into a 96‐deep wells plate, that is used for Ab staining. Consecutively, 18 μl of a mAb mix bound to different fluorescent labels was added to the 96‐wells plate. After 15 min of incubation, the blood was lysed using 335 μl of lysing reagents A and 100 μl of lysing reagent B. Lysing reagent A is a cyanide‐free lytic reagent that lyses red blood cells. Lysing reagent B slowed the reaction caused by reagent A and preserved the white blood cells for measurement in the flow cell. Finally, 100 μl of the prepared sample was aspirated for analysis.

A customized Ab mix was made specifically for this research purpose. This panel consisted of CD35‐FITC (clone J3.D3, Beckman Coulter, Miami, FL), CD16‐PE (clone 3G8, Beckman Coulter), CD62L‐ECD (clone DREG56, Beckman Coulter), CD11b‐PC5 (clone Bear1, Beckman Coulter), and CD10‐PC7 (clone ALB1, Beckman Coulter).

The .lmd data files were exported from the AQUIOS CL^®^ and imported into FlowJo^®^ analysis software (Tree Star Inc., Ashland, OH). The absolute total leukocyte count was extracted from the .pdf files generated by the AQUIOS CL^®^. The gating strategy is shown in Supplemental Figure 2. Hematology test reference ranges of the American board of internal medicine were used regarding absolute cell counts.^37^ Reference values of flow cytometry were determined with in the field laboratory with the use of healthy control non‐walkers. Granulocytes and monocytes were gated based on forward scatter and side scatter. Neutrophils and eosinophils in the granulocyte gate were identified based on positivity or negativity of CD16 expression. Neutrophil phenotypes were identified by the expression of CD16 and CD62L, as described in detail before.^28^ The gating strategy was checked for every individual by 2 independent researchers. The median fluorescent intensity (MFI) of gated granulocytes was used to describe the expression of CD35, CD11b, and CD10.

### 2.6 Statistical analysis

The statistical analyses were conducted in IBM SPSS^®^ Statistics for Windows version 26 (SPSS Inc., Chicago, IL, USA). Graphs were created with GraphPad Prism^®^ version 7 (GraphPad software inc., San Diego, CA, USA). Data were checked for normality with use of the D'Agostina & Pearson test and visual inspection of Q–Q plots. Statistical significance was defined as a *P*‐value < 0.05. Normally distributed data were presented as mean with sd. Non‐normally distributed data were presented as median with interquartile range (IQR).

Normally distributed data (only total WBC) were analyzed using repeated‐measures ANOVA to determine the effect of consecutive exercise days, followed by a Bonferroni post hoc multiple comparison correction. Non‐normally distributed data were tested by a Friedman's test, to evaluate the effect of consecutive exercise days with Dunn's post hoc multiple comparison correction.

Subgroup analyses were done on the neutrophil activation marker CD11b. A high CD11b at day 1 was defined as an MFI CD11b at day 1 above the IQR of the baseline MFI CD11b. A normal CD11b was defined as an MFI CD11b at day 1 within the IQR of baseline MFI CD11b. In this subgroup analysis, continuous data were shown as median (IQR) and the significance was tested with the Mann‐Whitney *U*‐test. Dichotomous data were shown as an absolute count (percentage), in which differences were tested with the Pearson Chi‐square test.

## 3 RESULTS

Demographics of the total study group are shown in Table 1. The study group consisted of 24 males and 15 females with a median age of 64 (58–70) years. The study group had a median BMI of 26 (24–30) kg/m^2^ and a median waist circumference of 96 (88–104) cm. The participants walked a distance of either 30 km (*n* = 19, 49%), 40 km (*n* = 19, 49%), or 50 km (*n* = 1, 2%). The median walking speed at day 1 was 4.4 (4.0‐4.8) km/h, 4.2 (3.8‐5.0) km/h at day 2, and 4.6 (4.0‐5.0) km/h at day 3. The median exercise intensity at day 1 was 65% (59–73%) of the maximum heart rate, being moderate exercise intensity.

**TABLE 1:** Demographics of the study group. Continuous data are shown as median (interquartile range) and dichotomous data are shown as absolute amount (percentage)

	(*N* = 39)
Age (years)	64 (58–70)
Male *n* (%)/female *n* (%)	24 (61%)/15 (39%)
Height (m)	1.77 (1.68–1.81)
Weight (kg)	82 (71–92)
Body mass index (kg/m^2^)	26 (24–30)
Waist circumference (cm)	96 (88–104)
Resting systolic blood pressure (mm Hg)	136 (130–149)
Resting diastolic blood pressure (mm Hg)	83 (74–90)
Resting heart rate (bpm)	69 (59–77)
Exercise intensity day 1 (%)	65 (59–73)
Distance walked (*n*, %)	
30 km	19 (49%)
40 km	19 (49%)
50 km	1 (2%)
Average speed (km/h)	
Day 1	4.4 (4.0–4.8)
Day 2	4.3 (3.8–5.0)
Day 3	4.6 (4.0–5.0)
Medical history (*n*, %)	
Cerebrovascular accident	7 (18%)
Asthma	7 (18%)
Deep venous thrombosis	6 (15%)
Psychiatric disorder	5 (13%)
Diabetes	5 (13%)
Atrial fibrillation	5 (13%)
Myocardial infarction	3 (8%)
Malignancy	3 (8%)
Medication use (*n* (%))	
Statin s	25 (64%)
Anticoagulants	12 (31%)
Ace‐2 inhibitors	9 (23%)
Diuretics	5 (13%)
Alfa blockers	5 (13%)
Beta blockers	5 (13%)
Proton pump inhibitors	5 (13%)
Antihistamines	2 (5%)
Other psychiatric drugs	6 (15%)
Other cardiovascular drugs	5 (13%)

### 3.1 Prolonged walking induced a systemic innate immune response after day 1

**FIGURE 2:**
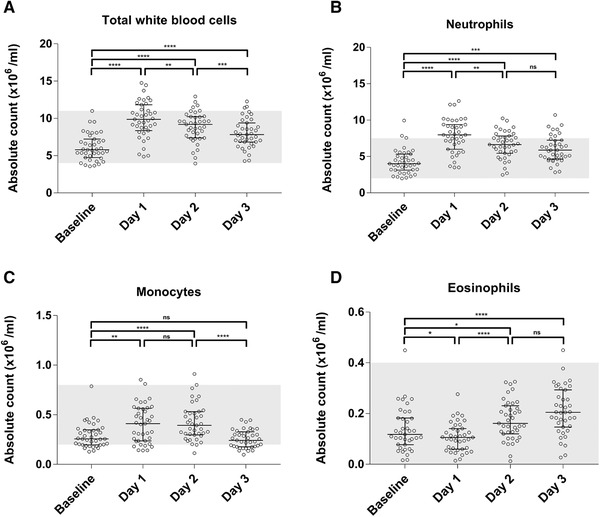
**The absolute count of total white blood cells (A), monocytes (B), and neutrophils (C) show a significant increase on day 1, followed by a decrease in the following days (*n* = 39)**. Eosinophils (**D**) show the opposite effect, a slight decrease on day 1 and after that a significant increase. The total white blood cell count was obtained from the cell counter in the automated flow cytometer. The percentage monocytes and granulocytes were gated base of forward versus side scatter. The percentage of neutrophils and eosinophils were determined by CD16^+^ and CD16^‐^ in the CD16 granulocyte histogram. Total white blood cells are shown as scatter plot with mean and sd, tested with ANOVA repeated‐measures with a Bonferroni post hoc multiple comparison correction. The rest of the data are presented as a scatter plot with median and interquartile range, tested with Friedman's test, with Dunn's post hoc multiple comparison correction. Reference values (grey area) show laboratory test reference ranges of the American board of internal medicine; ns, not significant; ^*^ *P* < 0.05; ^**^ *P* < 0.005; ^***^ *P* < 0.0005; ^****^ *P* < 0.0001

The absolute number of white blood cells, neutrophils, monocytes, and eosinophils are shown in Figure 2. The total white blood cell count increased after 1 day of walking (6.1 ± 1.8 × 10^6^/ml at baseline to 9.8 ± 2.4 × 10^6^/ml after 1 day of walking; *P* < 0.0001). This effect was mainly caused by an increase of neutrophilic granulocytes (4.0 (3.1–5.3) × 10^6^/ml at baseline to 7.9 (6.0–9.4) × 10^6^/ml after 1 day of walking; *P* < 0.0001). Monocytes also showed an increase after day 1 (0.35 (0.26–0.34) × 10^6^/ml to 0.41 (0.23–0.56) × 10^6^/ml; *P* < 0.005). Eosinophilic granulocytes show the opposite pattern of neutrophilic granulocytes, with a small but significant decrease after 1 day (0.11 (0.07–0.16) × 10^6^/ml to 0.10 (0.05–0.14) × 10^6^/mL; *P* < 0.05).

**FIGURE 3:**
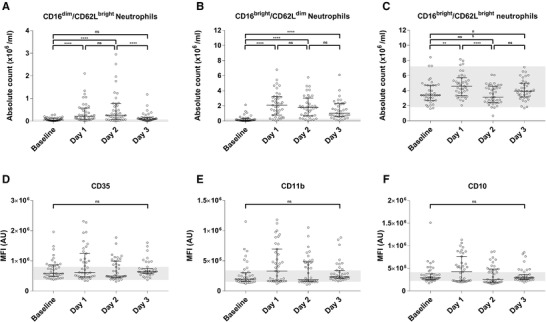
**A significant increase in absolute count of CD16^dim^/CD62L^bright^ neutrophils (A) and CD16^bright^/CD62L^dim^ neutrophils (B) was demonstrated on day 1 and 2 after repetitive, prolonged walking exercise (*n* = 39) and a decrease on day 3**. The CD16^bright^/CD62L^bright^ neutrophils (**C**) increase on day 1, decrease on day 2 and increase again on day 3. The median fluorescent intensity of neutrophil activation markers CD35 (**D**), CD11b (**E**), and CD10 (**F**) show no significant differences between the days. Neutrophils were gated based on CD16^+^ in the granulocyte population determined by forward versus side scatter. The 3 different neutrophil phenotypes (**A‐C**) were gated based on CD16/CD62L neutrophil plot. The MFI of activation markers (**D‐F**) was determined in the neutrophil population. Data are presented as a scatter plot with median and IQR, tested with Friedman's test and Dunn's post hoc multiple comparison correction. Reference values (grey area) show the interquartile range of healthy controls non‐walkers during the same event; ns, not significant; MFI, median fluorescent intensity; AU, arbitrary units; ^*^ *P* < 0.05; ^**^ *P* < 0.005; ^***^ *P* < 0.0005; ^****^ *P* < 0.0001

After 1 day, 2 additional neutrophil phenotypes were found increased in peripheral blood that are characteristic for systemic inflammation: CD16^dim^/CD62L^bright^ neutrophils and CD16^bright^/CD62L^dim^ neutrophils (Fig. 3A and B).^28^ The absolute number CD16^dim^/CD62L^bright^ neutrophils increased after 1 day: 0.04 (0.02–0.12) × 10^6^/ml at baseline to 0.21 (0.07–0.57) × 10^6^/ml after 1 day; *P* < 0.0001). Also the absolute number of CD16^bright^/CD62L^dim^ neutrophils was increased after 1 day (from 0.10 (0.03–0.35) × 10^6^/ml to 2.00 (0.77–3.24) × 10^6^/ml; *P* < 0.0001).

### 3.2 Normalization of systemic innate immune response after 2 and 3 days of walking

Repetitive prolonged walking exercise on consecutive days resulted in a partial normalization of tWBC from 9.8 ± 2.4 × 10^6^/ml on day 1 to 8.8 ± 2.0 × 10^6^/ml on day 2 (*P* < 0.005) and to 8.0 ± 1.9 × 10^6^/ml on day 3 (*P* < 0.0005). The decrease in tWBC count on day 2 was mainly the result of a partial normalization in neutrophil numbers (7.9 (6.0–9.4) × 10^6^/ml to 6.6 (5.4–7.8) × 10^6^/ml; *P* < 0.005). The decreased tWBC count on day 3 was a result of both a decrease in monocyte numbers (0.41 (0.23–0.56) × 10^6^/ml to 0.24 (0.17–0.32) × 10^6^/ml; *P* < 0.0001) and neutrophil numbers (6.6 (5.4–7.8) × 10^6^/ml to 5.8 (4.6–7.2) × 10^6^/ml; *P* = 0.816).

The 2 neutrophil phenotypes, CD16^bright^/CD62L^dim^ cells and CD16^dim^/CD62L^bright^ cells, associated with systemic inflammation were still present at day 2 (Fig. 3A and B). The decrease of total neutrophil count after day 2 of walking, was mainly the result of a decrease in CD16^bright^/CD62L^bright^ neutrophils (4.58 (3.33–5.70) × 10^6^/ml to 3.12 (2.41–4.57) × 10^6^/ml; *P* < 0.0001; Figure 3C). From day 2 to day 3 CD16^dim^/CD62L^bright^ neutrophils decreased (0.25 (0.06‐0.76) to 0.09 (0.05‐0.15) × 10^6^/ml; *P* < 0.0001). The CD16^bright^/CD62L^dim^ neutrophils slightly decreased after days 2 and 3.

### 3.3 Demonstration of an exponential relationship between CD62L and CD11b expression on neutrophils after repeated, prolonged walking

**FIGURE 4:**
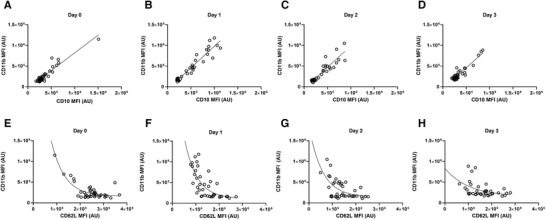
**Linear correlation between the median fluorescent intensity (MFI) of the neutrophil markers CD11b and CD10 was demonstrated over different days of prolonged, repeated walking**. Data are presented as a scatter plot with a linear regression line. At baseline, both CD11b and CD10 expression was low (**A**). At day 1, an increase was seen in both CD11b and CD10 with a linear correlation (*R* ^2 ^= 0.91; *P* < 0.0001; **B**). At days 2 and 3, both markers decreased but remain linear correlated (*R* ^2 ^= 0.83 and *R* ^2 ^= 0.87; *P* < 0.0001; **C‐D**). An exponential relationship between the MFI of CD62L and CD11b was found. Data are presented as a scatter plot with 1 phase decay exponential nonlinear regression. Day 0 showed one clear population of not activated participants with 4 activated outliers on the left (*R* ^2 ^= 0.76; A). At day 1, all participants had a decreased CD62L MFI. Only beyond a certain CD62L MFI decrease, CD11b increased as well (*R* ^2 ^= 0.53; B). At days 2 and 3, the variation in CD62L MFI remained, but the neutrophil activation maker CD11b decreased (**C‐D**). MFI, median fluorescent intensity; AU, arbitrary units

The MFIs of the neutrophil activation markers CD35, CD11b, and CD10 are shown in Figure 3D‐F. The expression of neutrophil activation markers CD11b, CD35, and CD10 all significantly correlated (*R* ^2 ^> 0.80; *P* < 0.0001; Figure 4A‐D). An exponential relationship was found between the most pronounced activation markers CD11b and CD62L, as shown in Figure 4E‐H. The increase in CD11b was associated with a decrease in CD62L in all cases, and thus with the presence of the CD16^bright^/CD62L^dim^ neutrophil subtype in the peripheral circulation. At days 2 and 3, there was a partial normalization back to baseline expression values of CD62L and CD11b.

### 3.4 Increased expression of CD11b on neutrophils was associated with slow walking speed and bad physical feeling

Participants with a high neutrophil CD11b expression after day 1 (*n* = 21) were compared to participants with normal CD11b expression after day 1, *n* = 18; Table 2). A total of 66% of the participants with increased CD11b expression walked 30 km compared to 34% who walked 40 or 50 km (*P* = 0.038). Despite the generally shorter walking distance, participants with an increased CD11b expression had a significantly slower walking speed on day 1 (4.1 km/h; *P* = 0.014), day 2 (4.1 km/h; *P* = 0.012), and day 3 (4.2 km/h; *P* = 0.003). Along with this significantly slower walking speed, participants with a high CD11b expression reported a worse physical feeling compared to participants with a normal CD11b expression on day 1 (*P* = 0.017) and day 3 (*P* = 0.022).

**TABLE 2:** Differences are shown between participants developing an increased level of the neutrophil activation marker CD11b at day 1 and participants who remain below the interquartile range (IQR) of baseline MFI CD11b values

	Normal CD11b at day 1 (*N* = 18)	High CD11b at day 1 (*N* = 21)	*P*‐value
Age	61 (56–69)	66 (60–70)	0.23
Male n (%) / female n (%)	12 (66%)/6 (33%)	12 (57%)/9 (43%)	0.54
Height (meter)	1.78 (1.70–1.81)	1.75 (1.66–1.81)	0.56
Weight (kg)	80 (70–87)	81 (69–94)	0.58
Body mass index (kg/m^2^)	25 (22–29)	26 (24–30)	0.34
Waist circumference (cm)	95 (88–101)	97 (87–105)	0.79
Resting systolic blood pressure (mm Hg)	137 (130–149)	135 (126–152)	0.81
Resting diastolic blood pressure (mm Hg)	85 (77–92)	80 (73–87)	0.15
Resting heart rate (bpm)	65 (58–73)	71 (59–77)	0.41
Exercise intensity day 1 (%)	61 (54–71)	65 (59–76)	0.25
Distance 30 km (n, %)	6 (33%)	13 (62%)	0.07
Distance 40/50 km (n, %)	12 (66%)	8 (38%)	0.038*
Walking speed day 1 (km/h)	4.6 (4.3–5.2)	4.1 (3.6–4.6)	0.014*
Walking speed day 2 (km/h)	4.7 (4.1–5.2)	4.1 (3.4–4.5)	0.012*
Walking speed day 3 (km/h)	4.9 (4.6–5.4)	4.2 (3.7–4.6)	0.003*
VAS feeling day 0	9.5 (8.2–9.8)	8.9 (8.1–9.5)	0.24
VAS feeling day 1	8.9 (7.2–9.5)	7.8 (6.2–8.3)	0.017*
VAS feeling day 2	8.7 (7.5–9.6)	8.8 (6.6–9.0)	0.13
VAS feeling day 3	8.9 (8.3–9.3)	8.0 (6.8–8.7)	0.022*
VAS effort day 1	5.5 (3.1–7.3)	5.4 (4.9–7.0)	0.74
VAS effort day 2	5.3 (2.4–6.9)	5.6 (2.7–6.9)	0.70
VAS effort day 3	5.2 (3.2–7.0)	5.4 (2.4–6.6)	0.79
VAS muscle pain day 0	0.2 (0.0–0.7)	0.3 (0.0–3.4)	0.44
VAS muscle pain day 1	1.0 (0.5–3.9)	2.3 (0.8–4.8)	0.32
VAS muscle pain day 2	2.1 (0.3–3.9)	2.0 (0.7–4.9)	0.47
VAS muscle pain day 3	1.9 (0.7–4.1)	1.2 (0.4–3.7)	0.53
Painkiller usage day 1 (n, %)	2 (11%)	6 (29%)	0.17
Painkiller usage day 2 (n, %)	3 (16%)	8 (38%)	0.13
Painkiller usage day 3 (n, %)	5 (28%)	9 (42%)	0.26

Continuous data are shown as median (IQR), the significance is tested with the Mann‐Whitney *U*‐test. Dichotomous data are shown as an absolute amount (percentage), the significance is tested with the Pearson chi‐square test; MFI, median fluorescent intensity; *significant.

## 4 DISCUSSION

To our knowledge, this is the first study in the literature that applied fully automated flow cytometry in a field laboratory to determine the effect of repetitive, prolonged walking exercise on consecutive days on the innate immune system. The study demonstrated the mobilization and activation of different neutrophil phenotypes in peripheral blood in response to prolonged exercise. The most pronounced immune response was found after day 1, followed by a partial normalization/adaptation in the days after that. Participants with a high neutrophil CD11b expression, indicative for neutrophil activation, were characterized by a higher degree of physiological stress imposed by the exercise load. Therefore, CD11b expression on neutrophils might be a valuable marker to monitor changes in the immune system after exercise.^38^

We used the neutrophil activation markers CD35, CD11b, and CD10, since they have been validated to demonstrate neutrophil activation in a fully automated flowcytometer.^25^ The complement receptors type 1 (CR1/CD35) and 3 (CR3/Mac1/CD11b) are known markers for neutrophil activation.^39^, ^40^ A reservoir of CR1 and CR3 receptors in neutrophils are present in cytoplasmic secretory vesicles, which are translocated to the plasma membrane upon cell activation.^39^, ^40^ The receptor CD10 is a known maturation marker and activation marker.^25^, ^41^, ^42^

The only other field study focusing on neutrophil phenotyping during exercise was done by Van Staveren et al.^27^ They investigated the expression of neutrophil markers of participants of an 8 days cycling tour with a mean daily distance of 160 km and 2300 altimeters.^27^ Blood sampling was performed in the morning before and after 4 and 8 days of cycling. This study showed an increased overall neutrophil count and an increased CD16^dim^/CD62L^bright^ and CD16^bright^/CD62L^dim^ after day 4 and 8 of cycling. Even though the same neutrophil phenotypes were studied it is challenging to compare the data to our study for several reasons: (i) our study was performed with a fully automated flow cytometer in a field laboratory whereas Van Staveren et al.^27^ collected, fixated, and froze the samples for later analysis in the laboratory; (ii) the first exercise data were obtained after 4 days, whereas this study determined the effects on neutrophils after 1, 2, and 3 days; and (iii) the exercise volume was not comparable. Nevertheless, we found a similar increase in CD16^dim^/CD62L^bright^ neutrophils and CD16^bright^/CD62L^dim^ neutrophils after 1 day of walking. Hereafter, a decrease in the number of these cells indicated adaptation of the innate immune system to a certain level of exercise. This is in contrast to van Staveren et al.,^27^ who showed a cumulative increase of systemic immune response after 4 and 8 days, which could be explained by the higher exercise intensity and load in their study, fitness level, and/or the sampling methods. However, one might speculate that low intensity prolonged walking exercise allows the immune system to adapt, while high‐intensity cycling at high altitude leads to an overreacting immune system preventing adaptation. In a previous study on repeated prolonged exercise, conducted during the same walking event, an increase in cytokines levels was shown after the first day and a normalization/adaptation the consecutive days.^43^ This leads to a similar conclusion regarding innate immune response, as we describe in this study.

After the first bout of prolonged exercise, several changes were visible in the neutrophil compartment in the peripheral blood. The CD16^dim^/CD62L^bright^ neutrophils were increased in the peripheral blood. These cells are mainly young neutrophils with a banded shaped nucleus released from the bone marrow.^44^ It is tempting to speculate that these neutrophils were mobilized from the bone marrow in response to damage‐associated molecular patterns (DAMPs) originating from the muscle damage in exercise. Furthermore, there was an increase of CD16^bright^/CD62L^dim^ neutrophils in all walking participants after day 1. This immune‐suppressive subset of neutrophils might be involved in immune regulation during physical exercise.^28^, ^29^ After days 2 and 3, a decrease in these neutrophil phenotypes was demonstrated indicative for adaptation of the innate immune system to exercise‐induced DAMPs.

All participants with a high neutrophil CD11b expression had an increase in number of CD16^bright^/CD62L^dim^ neutrophils as well. Therefore, the mere presence of high expression of the neutrophil activation marker CD11b also seems to correlate with the presence of immunosuppressive, CD16^bright^/CD62L^dim^ neutrophils in prolonged exercise.^28^ An increase of the immunosuppressive CD16^bright^/CD62L^dim^ neutrophils might be involved in the development of infectious illnesses during overtraining periods.

A limitation of this study is that a relatively elderly population was studied, which might not be representative of the general population. This age was in line with the mean age of participants who generally participate in these walking events.

With fully automated flow cytometry in a field laboratory, it is now possible to implement neutrophil phenotyping as a fast (20 min) and easily accessible test for studying the immune system in field settings. Future studies should also focus on high‐intensity exercise and should add more blood drawing moments to get a better understanding of the response of the innate immune system. For future studies in exercise immunology, it might be considered to integrate a fully automated flow cytometer in a moving mobile laboratory.

## 5 CONCLUSION

Repetitive, prolonged walking exercise on consecutive days results in an initial systemic innate immune response, followed by normalization/adaptation. An increase in neutrophil activation markers was associated with the presence of immunosuppressive neutrophils and possibly overtraining. Fully automated flow cytometry analysis of neutrophil phenotypes in a field laboratory might be a useful biomarker in the monitoring and tuning of, for example, exercise load during training periods of athletes.

## AUTHORSHIP

All authors made substantial contributions to the conception of the work. R.S., L.H., and C.C.G.W.B. worked on data acquisition. R.S., L.H., and C.B. performed the data analysis. All authors worked on the interpretation of data. R.S. made a substantial contribution to the drafting of the work. All authors made a substantial contribution to revising the work critically for relevant intellectual content. All authors approved the final version to be published. All authors agree to be accountable for all aspects of the work in ensuring that questions related to the accuracy or integrity of any part of the work are appropriately investigated and resolved.

## DISCLOSURE

The AQUIOS CL^®^ “load & go” flow cytometer is provided by the company Beckman Coulter Life Sciences, Miami, FL, USA. All authors declare that there are no other competing interests. This article was supported by a grant of the Netherlands Organization for Scientific Research (NWO) in the framework of the “Startimpulse” Dutch National Research Agenda.

## Supporting information

SUPPORTING INFORMATION

SUPPORTING INFORMATION
