# Supplementary figures and images for: Analysis of human neutrophil phenotypes as biomarker to monitor exercise‐induced immune changes
Source: J Leukoc Biol. 2020 Sep 6;109(4):833–42. doi: 10.1002/JLB.5A0820-436R (PMC8048637; doi:10.1002/JLB.5A0820-436R)

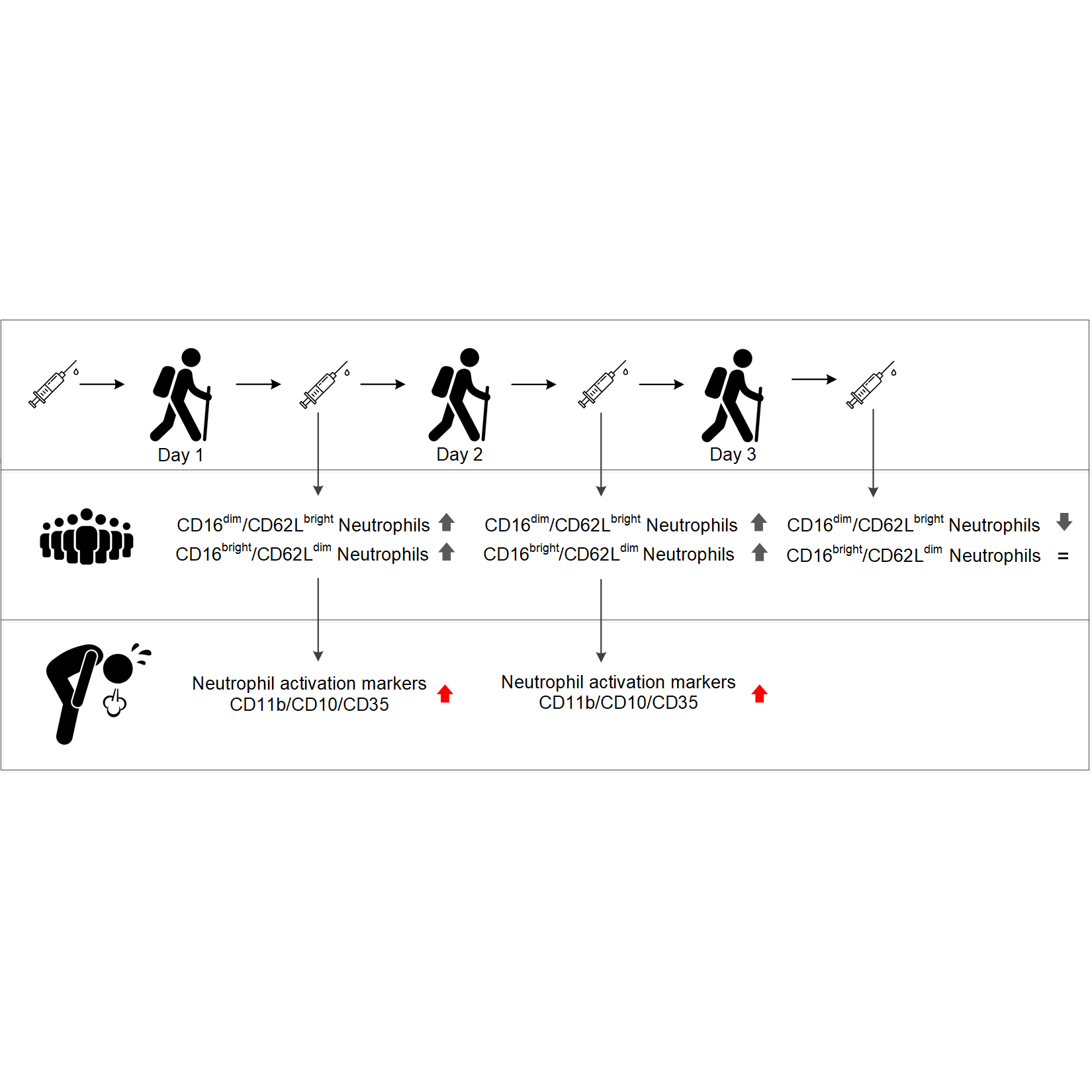

Supplement: Supplementary file 1 — SUPPORTING INFORMATION [file JLB-109-833-s001.tif]

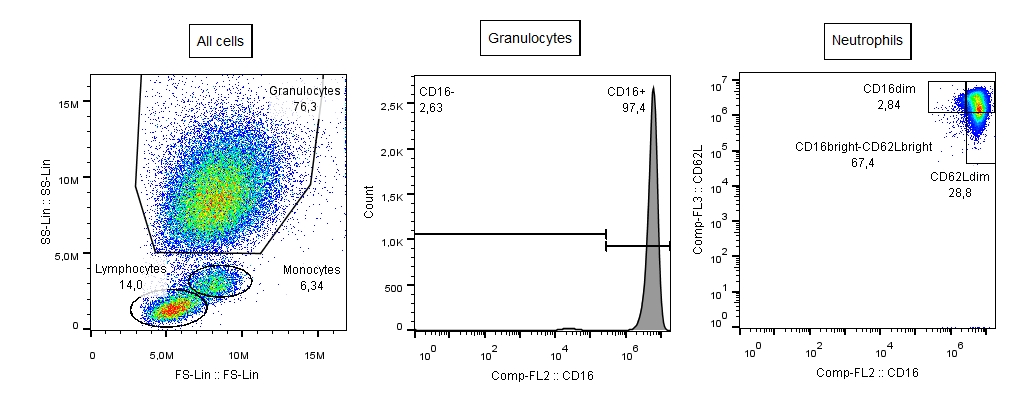

Supplement: Supplementary file 2 — SUPPORTING INFORMATION [file JLB-109-833-s002.jpg]
